# International classification of external causes of injury: a study on its content coverage

**DOI:** 10.1186/s12911-021-01515-9

**Published:** 2021-05-13

**Authors:** Leila Ahmadian, Fatemeh Salehi, Shabnam Padidar

**Affiliations:** 1 Medical Informatics Research Center, Institute for Futures Studies in Health, Kerman University of Medical Sciences, Haftbagh Highway, Kerman, 7616911313 Iran; 2 Health Human Resources Research Center, School of Management and Information Sciences, Shiraz University of Medical Sciences, Shiraz, Iran; 3 Emamreza Hospital, Mashhad University of Medical Sciences, Mashhad, Iran; 4 School of Management and Medical Information, Kerman University of Medical Sciences, Kerman, Iran

**Keywords:** International classification of external causes of injury (ICECI), Etiology, Injury prevention

## Abstract

**Background:**

Injuries are a major health issue worldwide and their prevention requires access to accurate statistics in this regard. This can be achieved by classifying the collected data using the international classification systems. This study aimed at investigating the content coverage rate of the International Classification of External Causes of Injury (ICECI) regarding the external causes of injury in a hospital.

**Methods:**

This cross-sectional descriptive-analytical study was performed on 322 injured individuals visiting the emergency unit of a hospital which is the biggest truma center in the southeast of Iran. The required data were collected via a designed questionnaire by the researcher visiting the Emergency ward. The collected data were encoded based on the ICECI textbook by two encoders. Their agreement rate was calculated using the Kappa estimate of agreement. The content coverage of the classification system and the degree of completeness of the required data for encoding in the patients’ records were measured. Data were analyzed by the SPSS software, ver 19.

**Results:**

The findings showed that 70% of the external causes of injury were covered by ICECI. Among the 322 cases, 138 (43%) had been referred due to a car crash. The injured were mostly drivers of land transport vehicles who had been unintentionally involved in a car crash. The least mechanism for injury was bite injury with 5 (2%). ICECI was capable of classifying 92% of the data related to the external causes of the injuries. The most un-covered data has belonged to the "activity when injured" axis (n = 18). Lack of precise data recording in the medical records resulted in missing data about at least one of the axis of the external causes in most records.

**Conclusion:**

The findings of the present study showed that ICECI has good content coverage for encoding the external causes of injuries**.** Before implementing ICECI for encoding the external causes of injuries, it is required to train clinicians regarding how to document all aspects of an injury incidence.

## Background

Injuries from external causes kill more than five million people a year worldwide. This causes 9% of world mortality. Millions of people harmed from them, require hospitalization or outpatient and emergency care [1, 2]. Injuries and violence have been neglected from the global health agenda for many years, despite being predictable and largely preventable [3]. Such injury events impose a major burden on both the government and the community. To reduce the undesired effects of such incidents, the design and performance of injury prevention strategies with access to accurate statistics seem essential [4–7]. This cannot be done except by using data on the causes of injuries that are correctly and thoroughly coded and used by policymakers to develop regulations and rules [8, 9]. For encoding in the field of health, various international classification systems are available each of which has a different application. The International Classification of External Causes of Injury (ICECI) is an example; it is a multi-axis classification system with hierarchical codes. Since this system focuses on all details of the incident, the resulting data can be highly beneficial in injuries prevention [10, 11].

A few studies have been done on the prevention of injury by applying this coding system in other countries. In a study done by Ahn the external causes of severe pediatric injuries were classified using ICECI [5]. In a previous study, an international spinal cord injury prevention system was developed using the ICECI [4]. In this study, the validity and reliability of ICECI were evaluated in a mixed set of trained and untrained coders [4]. One study evaluating software that collects data about burn injuries using ICECI in the United States [7]. Another study evaluating the causes of injuries and traumas experienced during school sports and recreation [12]. Studies done until now using this classification system focused on one type of injury or classified the external causes of the injuries in a group of patients.

### The ICECI

Since 1980, a lot of criticism has been made regarding the ICD deficiencies such as lack of codes on the nature of injuries, and lack of logic and flexibility of this coding system for external causes of injuries. In April 1998, the WHO introduced an experimental system in the form of a list of tables as the International Classification of External Causes of Injury (ICECI). The last electronic version of this classification was published in July 2004 [13, 14]. This classification is used as a tool in the data management of external causes of injury. The aim for its design was to aid professionals and researchers in the statistical tracking and prevention of injury; it was developed to be applied in centers by using their recorded data the statistical reports of injuries could be provided.

This system has certain structural and conceptual characteristics that distinguishes it from the International Classification of Diseases (ICD). Conceptually, it is based on a clear and obvious model of events and incidents. Regarding its structure, it is a multi-axis, multipart classification system with hierarchical codes [15]. This system is provided as an alphanumeric list and has two modules: the "core module" and "additional modules". The main part is completed for all types of injuries and includes 7 axes in the new revision as follows: 1. the role of human intent, 2. mechanism of injury, 3. objects/substances producing injury, 4. place of occurrence, 5. activity when injured, 6. Use of alcohol and 7. use of (other) psycho-active drugs. The "additional modules" include 5 parts: violence, transport, place, sports, and occupation. The codes of this section are used when the codes in the core module do not represent the details of the event [15].

In the present study, a wide range of injuries have been investigated and no restriction such as studing a specific age group of patients has been considered. Collecting the data related to the external cause of injuries along with their classification and coding based on the ICECI can provide accurate statistics to health policymakers. So this study aimed to investigate the content coverage rate of the International Classification of External Causes of Injury (ICECI) regarding the external causes of injury in a large trauma center in the southeast of Iran.

## Methods

In this cross-sectional descriptive-analytical study the external causes of injuries in Shahid Bahonal Hospital, Kerman, Iran were classified based on the ICECI. This hospital is the largest trauma center in the southeast of Iran. The study population consisted of all injured patients visiting this hospital from Jan to March 2016. Due to the large study population (n = 2004), the Cochrane sampling method [16] were used resulting in a study sample of 322 cases (*p* = 0.5, q = 0.5, z = 1.96). The patients selected for inclusion in the study with simple random sampling. To recruit the sample, one of the researchers visited the hospital in different hours during three hospital rounds and randomly chose the patient number of admitted patients.

The required data were collected via a designed questionnaire by the researcher visiting the Emergency ward. The questionnaire consisted of demographic data (age, sex, educational status, and occupation), present and past medical history, and the external causes of injuries precisely according to the axes of the ICECI. Content validity was measured by CVR and CVI and confirmed by 3 medical informatics specialists and two health information management experts. The reliability of the questionnaire was evaluated by a test–retest procedure on a sample of 15 patients; furthermore, the questionnaire achieved a Cronbach’s alpha of 0.81. The researcher visited the emergency unit three times per day, at the morning, afternoon, and night to collect the essential data from the injured patients. Following patient transfer to the emergency unit and after receiving the emergent primary care in case of consciousness and a stable condition the researcher spoke directly to the patients whereas in unstable and unconscious patients the patients’ relatives were interviewed to complete the questionnaire. In the case of no patients’ relatives, the questionnaire was completed after the patient's partial recovery.

For data collection on the present illnesses and the past medical history, the patient's records were also studied. After completing data collection, two encoders (health IT experts) encoded the data form based on the ICECI. To increase the credibility of the results, coding was done independently by two experts. In case of disagreement between the assigned codes, it was resolved through discussion by the encoders and subsequently via consultation with an expert (Ph.D. in Medical Informatics). In this approach the assigned codes were initially extracted from the main text of the book and in case of not being covered in the main text, the encoder referred to the "additional modules" to assign the selected code to the diagnoses. Data encoding was done without delay so that in the event of missing data the additional information could be obtained from the patients. The accuracy and correctness of the encoding were checked by a coding expert.

In addition, the data collected through the questionnaire were compared with the data recorded in the patients' records. The purpose of this comparison was to examine the extent of which data on the causes of injuries were recorded in medical records.

The collected data were analyzed by the SPSS software, ver. 19. The frequency of mechanisms of injury, objects involved in the injuries, and places of injuries was presented based on age, sex, and educational status and occupation. The relationship between the patients’ demographics and mechanisms of injuries was tested by the chi-square test. The significant level set to less than 0.05. Moreover, the coverage rate of the main module and the additional module of the classification system were calculated.

Written informed consent was obtained from each participant before study entrance and all collected data were regarded as confidential. Ethical approval was received from the Kerman University of Medical Sciences. (Ethical number: IR.KMU.REC.1394.328).

## Results

In total 322 injured patients visiting the emergency unit were studied. Interviewing patients to collect the required data lasted at a maximum of 10 min. The researcher also spent 5 min to review the medical records of the included patients. Reviewing medical records by reviewers revealed that none of the medical records included all aspects of the causes of the injuries.

By calculating a Kappa coefficient of 0.88, an acceptable agreement was achieved between the two encoders in encoding the data which increases the credibility of this study. The disagreement between two coders mostly occurred in coding car crash as the mechanism of the injuries.

According to Table 1, the most common mechanism causing injury was car crash in 138 (43%) and the least common were bites in 5 (2%). The mechanism of injury had a wider spectrum in males compared to females; moreover, the rate of injury was higher in youth (aged 18–34 years), self-employed individuals, and those with an under diploma degree (Table 1). The results of the chi-square test showed that there is a significant difference between the mechanisms of the injuries and sex and age of the participants. The car crash was more frequent among male and youth individuals.

**Table 1:** Frequency (%) of the injury causing mechanism based on sex, age, educational level and occupation

Variable	Car crash	Cuts	Fight	Bites	Fall down	Fall from height	Crushing	Others	*p* value
Sex									
Male	105 (44.11)	28 (11.76)	20 (8.40)	5 (2.10)	34 (14.28)	19 (7.98)	6 (2.52)	21 (8.82)	0.004
Female	33 (39.28)	7 (8.33)	2 (2.38)	0 (0)	32 (38.09)	2 (2.38)	3 (3.57)	5 (5.95)	
Age									
Child	18 (30.50)	5 (8.47)	0 (0)	1 (1.69)	21 (35.59)	2 (3.38)	7 (11.86)	5 (8.47)	0.012
Adolescent	18 (52.94)	3 (8.82)	4 (11.76)	1 (2.94)	6 (17.64)	1 (2.94)	0 (0)	1 (2.94)	
Youth	69 (53.48)	15 (12)	15 (11.62)	2 (1.55)	11 (8.52)	8 (6.20)	1 (0.77)	8 (6.20)	
Middle-aged	27 (34.61)	12 (15.38)	3 (3.84)	1 (1.28)	18 (23.07)	7 (8.97)	1 (1.28)	9 (11.53)	
Elderly	6 (27.27)	0 (0)	0 (0)	0 (0)	10 (45.45)	3 (13.63)	0 (0)	3 (13.63)	
Educational level									
Illiterate	12 (24.00)	1 (2.00)	3 (6.00)	1 (2.00)	20 (40.00)	2 (4.00)	6 (12.00)	5 (10.00)	0.538
Student	23 (44.23)	7 (13.46)	2 (3.84)	0 (0)	14 (26.92)	2 (3.84)	2 (3.84)	2 (3.84)	
< Diploma	67 (44.37)	15 (9,93)	14 (9.27)	2 (1.32)	22 (14.56)	15 (9.93)	1 (0.66)	15 (9.93)	
University degree	36 (52.17)	12 (17.39)	3 (4.34)	2 (2.89)	10 (14.49)	2 (2.89)	0 (0)	4 (57.97)	
Occupation									
Unemployed	18 (52.29)	0 (0)	1 (2.94)	0 (0)	9 (26.47)	3 (8.82)	0 (0)	3 (8.82)	0.170
Self-employed	53 (43.80)	15 (12.39)	14 (1157)	3 (2.47)	9 (7.43)	12 (9.91)	2 (1.65)	13 (10.74)	
Housewife	13 (36.11)	4 (11.11)	1 (2.77)	0 (0)	15 (41.66)	1 (2.77)	0 (0)	2 (5.55)	
Employed	14 (56.00)	5 (2.00)	1 (4.00)	0 (0)	1 (4.00)	2 (8.00)	0 (0)	2 (8.00)	
Student	34 (44.15)	10 (12.98)	5 (6.49)	1 (1.29)	21 (27.27)	2 (2.59)	2 (2.59)	2 (2.59)	
Child	6 (19.35)	1 (3.22)	0 (0)	1 (3.22)	11 (35.48)	4 (12.90)	4 (12.90)	4 (12.90)	
Total	138 (42.85)	35 (10.86)	22 (6.83)	5 (1.55)	66 (20.49)	21 (6.52)	9 (2.79)	26 (8.07)	

Given the objects causing injury, land-transport vehicles had the highest prevalence (38%); the objects with least injury were children's toys, mobile machinery, sports equipments, and food and drinks (Table 2). According to the collected data regarding the place of incident, public transportation had the highest risk of injury as revealed in 162 (50%) cases (Table 3).

**Table 2:** Frequency (%) of objects that cause accident by gender, age, education and occupation*

Variable	Vehicles	Home furnishings	Home Appliances	Kitchenware	Personal belongings	Work-related equipment	Weapons	Construction components	Construction components	Ground level	Natural and industrial materials	Non-medical chemicals	Others	Unknown	Total
Sex															
Female	27 (32.14)	3(3.57)	2 (2.38)	4(4.76)	1(1.19)	2(2.38)	0	1(1.19)	6(7.14)	10(11.90)	5(5.95)	3(3.57)	4(4.76)	15(17.85)	84(100)
Male	94(39.49)	4(1.68)	1(0.42)	16(6.72)	1(0.42)	20(8.40)	4(1.68)	15(6.30)	14(5.88)	15(6.30)	11(4.62)	0	21(8.82)	19(7.98)	238(100)
Age															
Child	2(3.38)	2(3.38)	2(3.38)	2(3.38)	0	0	0	2(3.38)	7(20.58)	7(20.58)	5(8.47)	0	4(6.77)	7(20.58)	59(100)
Adolescent	16(47.05)	0	0	5(14.70)	1(2.94)	0	0	1(2.94)	3(8.82)	1(2.94)	0	0	2(5.88)	5(14.70)	34(100)
Youth	57(44.18)	1(0.77)	0	11(8.52)	0	12(9.30)	3(2.32)	8(6.20)	3(2.32)	8(6.20)	6(4.65)	0	11(8.52)	7(5.42)	129(100)
Middle-aged	22(28.20)	4(5.12)	1(1.28)	2(2.56)	1(1.28)	7(8.97)	1(1.28)	5(6.41)	5(6.41)	6(7.69)	4(5.12)	3(3.84)	8(10.25)	8(10.25)	78(100)
Elderly	6(27.27)	0	0	0	0	3(13.63)	0	0	2(9.09)	3(13.63)	1(4.54)	0	0	7(31.81)	22(100)
Educational level															
Illiterate	14(28.00)	1(2.00)	2(4.00)	2(4.00)	0	1(2.00)	1(2.00)	1(2.00)	5(10.00)	5(10.00)	2(4.00)	1(2.00)	4(8.00)	10(20)	50(100)
Student	23(44.23)	1(1.92)	0	4(7.69)	1(1.92)	0	0	1(1.92)	6(11.53)	4(7.69)	3(5.76)	0	3(5.76)	6(11.53)	52(100)
< Diploma	56(37.08)	2(1.32)	0	10(6.62)	1(0.66)	13(8.60)	3(1.98)	9(5.96)	7(4.63)	11(7.28)	8(5.29)	2(1.32)	14(9.27)	12(7.94)	151(100)
University degree	28(40.57)	3(4.34)	1(1.44)	4(5.79)	0	8(11.59)	0	5(7.24)	2(2.89)	5(7.24)	3(4.34)	0	4(5.79)	6(8.69)	69(100)
Occupation															
Unemployed	14(41.17)	2(5.88)	0	1(2.94)	0	2(5.88)	0	1(2.94)	2(5.88)	5(14.70)	0	1(2.94)	1(2.94)	5(14.70)	34(100)
Self-employed	47(38.84)	1(0.82)	1(0.82)	8(6.61)	0	14(11.57)	4(3.30)	6(4.95)	4(3.30)	7(5.78)	7(5.78)	0	12(9.91)	7(5.78)	121(100)
Housewife	11(30.55)	0	0	3(8.33)	1(2.77)	1(2.77)	0	1(2.77)	3(8.33)	3(8.33)	1(2.77)	2(5.55)	3(8.33)	7(19.44)	36(100)
Employed	10(40.00)	2(8.00)	0	1(4.00)	0	3(12.00)	0	2(8.00)	0	0	3(12)	0	1(4.00)	3(12.00)	25(100)
Student	32(41.58)	1(1.29)	0	6(7.79)	1(1.29)	2(2.59)	0	5(6.49)	7(9.09)	8(10.38)	3(3.89)	0	5(6.49)	7(9.09)	77(100)
Child	7(24.13)	1(3.44)	2(6.89)	1(3.44)	0	0	0	1(3.44)	4(13.79)	2(6.89)	2(6.89)	0	3(10.34)	5(17.24)	29(100)

^*^Mobile machinery, Sport equipment, Children's toys and Foods or drinks in total had frequency less than 4 and were not listed in the table

**Table 3:** Frequency (%) of accident place in which the injuries occurred by gender, age, education and occupation

Variable	Home	Residential areas	Educational institutes	Sport centers	Public transport sites	Other public transport sites	Industrial or construction sites	Fields of production or farms	Recreational-cultural sites	Shopping centers	Suburbs	Other Place	Unknown place	Total
Sex														
Female	33(39.28)	0	4(4.76)	1(1.19)	39(46.42)	1(1.19)	0	0	2(2.38)	2(2.38)	1(1.19)	0	1(1.19)	84(100)
Male	52(21.84)	3(1.26)	9(3.78)	2(0.84)	123(51.68)	5(2.10)	16(6.72)	2(0.84)	2(0.84)	6(2.52)	5(2.10)	2(0.84)	11(4.62)	238(100)
Age														
Child	20(35.71)	0	8(14.28)	0	21(37.5)	0	0	1(1.78)	2(3.57)	0	3(5.35)	0	1(1.78)	56(100)
Adolescent	5(14.70)	0	4(11.76)	0	19(55.88)	1(2.94)	0	0	0	0	2(5.88)	1(2.9)	2(5.88)	34(100)
Youth	24(18.60)	2(1.55)	0	2(1.55)	76(58.91)	3(2.32)	11(8.52)	0	0	3(2.32)	1(0.77)	1(0.77)	6(4.65)	129(100)
Middle-aged	26(33.33)	0	1(1.28)	1(1.28)	34(43.58)	2(2.56)	4(5.12)	1(1.28)	1(1.28)	5(6.41)	0	0	3(3.84)	78(100)
Elderly	8(36.36)	1(4.54)	0	0	11(50.00)	0	1(4.54)	0	1(4.54)	0	0	0	0	22(100)
Educational level														
Illiterate	21(42.00)	0	2(4.00)	0	18(36.00)	0	0	1(2.00)	3(6.00)	1(2.00)	3(6.00)	0	0	50(100)
Student	11(21.15)	0	10(19.23)	0	24(46.15)	0	0	0	0	0	3(5.76)	1(1.92)	3(5.76)	52(100)
< Diploma	37(24.50)	2(1.32)	1(0.66)	2(1.32)	78(51.65)	4(2.64)	13(8.60)	1(0.66)	1(0.66)	5(3.31)	0	0	7(4.63)	151(100)
University degree	16(23.18)	1(1.44)	0	1(1.44)	41(59.42)	2(2.89)	3(4.34)	0	0	2(2.89)	0	1(1.44)	2(2.89)	69(100)
Occupation														
Unemployed	7(20.58)	2(5.88)	1(2.9)	0	19(55.88)	1(2.9)	1(2.9)	0	2(5.88)	1(2.9)	0	0	0	34(100)
Self-employed	20(16.52)	1(0.82)	1(0.82)	2(1.65)	62(51.23)	4(3.30)	14(11.57)	1(0.82)	0	6(4.95)	1(0.82)	1(0.82)	8(6.61)	121(100)
Housewife	16(44.44)	0	0	0	18(50.00)	1(2.70)	0	0	0	1(2.70)	0	0	0	36(100)
Employed	9(36.00)	0	0	0	15(12.39)	0	1(4.00)	0	0	0	0	0	0	25(100)
Student	17(22.07)	0	10(2.98)	1(1.29)	40(51.94)	0	0	1(1.29)	0	0	3(3.89)	1(1.29)	4(5.19)	77(100)
Child	16(55.17)	0	1(3.44)	0	8(27.58)	0	0	0	2(6.89)	0	2(6.89)	0	0	29(100)

More than half of the injured patients (58%) used their personal vehicles to get to the Bahonar Hospital Emergency unit and did not contact emergency call services. The mean duration between the incident occurrences to hospital arrival was 46 min + 13 s.

Regarding the "activity when injured" axis, 85 (26%) injured patients were involved in other activities such as walking, jogging, sitting, etc. during the incident; The exact rate of different activities when injured is presented in Table 4. In addition, when studying all injuries, they had mostly occurred unintentionally (n = 292, 91%) whereas only 22 (7%) were done intentionally.

**Table 4:** The activity of the injured cases during the incident

Activity	Frequency	Percentage
Doing work with payment	38	11.80
Doing work without payment	4	1.24
Sports and exercise	1	0.31
Games and entertainment	42	13.04
Vital activities	7	2.17
Pedestrian	34	10.55
Driver	69	21.42
Passenger	36	11.18
Other activities	85	26.39
Unknown	6	01.86
Total	322	100

On the other hand, only one of the injured patients and 2 of the other counterparts involved in an incident had used alcohol before the incident; the same figures were 22 (7%) and 2(0.6%) for psycho-active drugs.

**Fig. 1:**
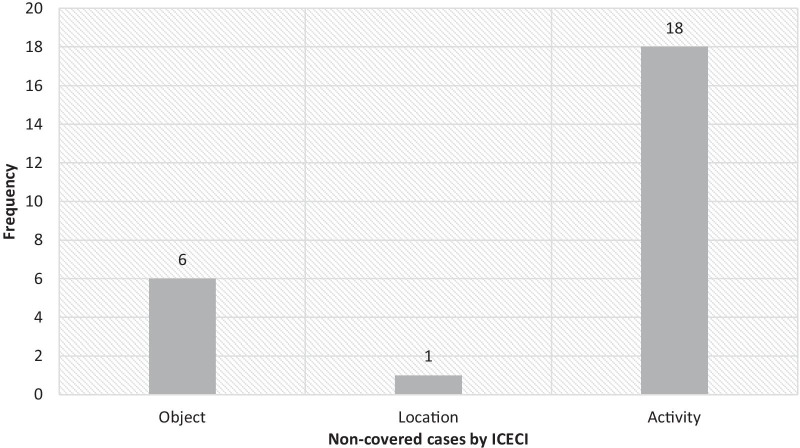
Frequency of non-covered data by ICECI

Furthermore, the data describing the external causes of injuries in around 25 (8%) patients in Shahid Bahonar Emergency Unit were not classifiable by the ICECI; the details have been presented in Fig. 1.

As the ICECI textbook consists of two parts, the "core module" and "additional modules", in which the first part focuses on the high level of granularity and the second part provides detailed granularity, the content coverage of the second part was higher. For instance, in the core module, the place of injury indicates e.g. home, whereas the second part emphasis on the details such as which room at home; accordingly the content coverage rate of the core module was 47% (n = 152) and the additional modules was 53% (n = 170).

In general, only in one (0.3%) of the studied patients' records, all the seven axes of injury were recorded whereas in the majority of records the data on at least one axis was missing. In addition, over 50% of the data collected by the researcher was obtained from the patient's relatives as the patients were either a child or not stable enough to respond (Table 5).

**Table 5:** Frequency percentage coverage rate of the ICECI according to the data not recorded in Shahid Bahonar Hospital

Non-covered cases by ICECI	Frequency	Percentage
Objects	6	1.86
Place	1	0.31
Activity	18	5.59
Total	25	7.76

## Discussion

The findings of the present study showed that ICECI has good content coverage for encoding the causes of external injuries; this has also been approved in other studies in this respect [9]. The "additional modules" of this classification has provided the encoders with more detailed data and the possibility to describe the external causes of the injuries more specifically [15]. In this study, to obtain the data regarding the causes of the injuries besides interviewing patients the patient medical records were also thoroughly studied. This review revealed the deficiencies in data recording in medical records regarding the external causes of injuries which highlights the need for further research and planning concerning this issue.

Incomplete recording of data in medical files regarding the causes of injuries could cause certain challenges in the functional application of this system as the optimal usage of this classification system for preventive purposes requires the precise recording of such data in the medical files of patients.

In this study, the mean time interval between the incident and the patient's arrival at the medical center was much longer than the standard time (< 9 min). The secure transfer of injured cases by ambulances to medical centers in the standard time is of great importance for reducing the mortality rate [17–19].

As described in the methodology, in this study the data were encoded by two health information technology experts.The mean coding time to encode all aspects of the external causes of the injury was 3 min. As this time is not long in comparison to the valuable data which can be obtained through these encodings, it is recommended to code the external causes of injuries using this classification system continuously. As determining the external causes of the injuries help policymakers to plan for preventing programs.

Regarding the results of the current study, car crashes were the most common mechanism of injury. Therefore, the presence of controlling tools and appropriate urban-road infrastructures can result in a reduced number of unintentional car crashes. Although the content coverage of the ICECI was good in our study and more than 90 percent of the information could be codded by this terminology, however, there was some information that can not be coded by this terminology and not all causes of injuries can be covered by ICECI. Therefore, like other terminologies, there are deficiencies in this terminology that should be addressed in future revisions. Moreover, with current documenting data in the medical records, it is not possible to encode all aspect of the injuries using ICECI. Therefore, to encode the data using this terminology it is recommended to train the clinicians in documenting the required data before the implementation of this terminology.

### Strengths and limitation

One of the strengths of this study is investigating the causes of all kinds of the injuries that patients faced during the study period. This made it possible to study different parts of the ICECI system comprehensively. It is worth noting that this is the only study conducted in Iran on the classification of external causes of multiple injuries by the ICECI system. The other research performed in this respect have focused on a single injury such as spinal injuries, head traumas in children, burn injuries [4, 5, 7, 12]. Other strengths include encoding information by two coders, which increases the validity of the results. Additionally, in this study, in addition to collecting information from the patient and his / her relative, the medical records of the patients were also thoroughly investigated, which led to the discovery of possible deficiencies in the records.

This study had two limitations. The first limitation was the lack of cooperation of injured patients with the researcher. Moreover, inaccurate information can also be provided to the researcher, especially regarding alcohol and psychotropic substances, which may partly lead to invalidation of the results in this part of the study. This kind of information in some cases is not provided to the clinicians to document in the medical records.

## Conclusion

The findings of the present study showed that ICECI has good content coverage for encoding the causes of external injuries**.** Complete and accurate recording of the external causes of injuries can help coders to encode the data more accurately and health policymakers to prevent injuries. Therefore, before implementing ICECI for encoding the external causes of injuries, it is required to train clinicians regarding how to document all aspects of an injury incidence. Successful clinical documentation facilitate the accurate representation of a patient’s clinical status that translates into coded data. Moreover, as some of the gathered data regarding the external causes of injuries could not be classified by the ICECI system, the findings of this study in addition to identifying the deficiencies of this system, can help its developers to extend it in the future revisions.

## Data Availability

The datasets available from the corresponding author on reasonable request.

## Appendix 1: A list of external causes of injury in patients referred to the emergency department of Kerman Bahonar Hospital

**Table:**

Demographic information							
	Occupation		Educational level		Age		Sex
Current and past history of diseases							
							Current disease
							History of diseases
							History of previous injuries
							The nature of the injury
Relevant codes based on ICECI system		External causes of injuries					
							Mechanism of injury
							Intention to cause harm
							The tools causing injury
							The sites in which the injuries occurred
							The activity of the injured cases during the incident
							Alcohol use when the injury occurs
							Use of other psychoactive substances in the event of injury
Encoding time							How to get the patient to the hospital
							The interval between the accident and the patient's arrival at the hospital
Death		Referral to another center		Recovery		Partial recovery	Length of stay and outcome of treatment

## Appendix 2: List of items not covered by the ICECI system

**Table:**

Items not covered by ICECI system	
Walking, arguing, sitting, running, cleaning the house, washing dishes, walking, moving household items, talking, installing curtains, praying, flagging, jumping, dancing, getting off the car, crossing the street, up Going up the ladder and making toys	Activity
Hard body, bridge, guard rail, electrical equipment, freight handle and rebar	Objects
Ruined house	Place

## Abbreviation

ICECI: International classification of external causes of injury
